# LipoParticles: Lipid-Coated PLA Nanoparticles Enhanced In Vitro mRNA Transfection Compared to Liposomes

**DOI:** 10.3390/pharmaceutics13030377

**Published:** 2021-03-12

**Authors:** Camille Ayad, Pierre Libeau, Céline Lacroix-Gimon, Catherine Ladavière, Bernard Verrier

**Affiliations:** 1 UMR 5305: Laboratoire de Biologie Tissulaire et d’Ingénierie Thérapeutique, Institut de Biologie et Chimie des Protéines, CNRS/Université Claude Bernard Lyon 1, 7 passage du Vercors, CEDEX 07, 69367 Lyon, France; pierre.libeau@ibcp.fr (P.L.); celine.fish@free.fr (C.L.-G.); 2 UMR 5223: Ingénierie des Matériaux Polymères, CNRS/Université Claude Bernard Lyon 1, Domaine Scientifique de la Doua, Bâtiment POLYTECH, 15 bd André Latarjet, CEDEX, 69622 Villeurbanne, France

**Keywords:** LipoParticles, mRNA vaccines, liposomes, transfection, nucleic acids, cell-penetrating peptides, delivery systems, nanoparticles

## Abstract

The approval of two mRNA vaccines as urgent prophylactic treatments against Covid-19 made them a realistic alternative to conventional vaccination methods. However, naked mRNA is rapidly degraded by the body and cannot effectively penetrate cells. Vectors capable of addressing these issues while allowing endosomal escape are therefore needed. To date, the most widely used vectors for this purpose have been lipid-based vectors. Thus, we have designed an innovative vector called LipoParticles (LP) consisting of poly(lactic) acid (PLA) nanoparticles coated with a 15/85 mol/mol DSPC/DOTAP lipid membrane. An in vitro investigation was carried out to examine whether the incorporation of a solid core offered added value compared to liposomes alone. To that end, a formulation strategy that we have named particulate layer-by-layer (pLbL) was used. This method permitted the adsorption of nucleic acids on the surface of LP (mainly by means of electrostatic interactions through the addition of LAH4-L1 peptide), allowing both cellular penetration and endosomal escape. After a thorough characterization of size, size distribution, and surface charge— and a complexation assessment of each vector—their transfection capacity and cytotoxicity (on antigenic presenting cells, namely DC2.4, and epithelial HeLa cells) were compared. LP have been shown to be significantly better transfecting agents than liposomes through pLbL formulation on both HeLa and DC 2.4 cells. These data illustrate the added value of a solid particulate core inside a lipid membrane, which is expected to rigidify the final assemblies and makes them less prone to early loss of mRNA. In addition, this assembly promoted not only efficient delivery of mRNA, but also of plasmid DNA, making it a versatile nucleic acid carrier that could be used for various vaccine applications. Finally, if the addition of the LAH4-L1 peptide systematically leads to toxicity of the pLbL formulation on DC 2.4 cells, the optimization of the nucleic acid/LAH4-L1 peptide mass ratio becomes an interesting strategy—essentially reducing the peptide intake to limit its cytotoxicity while maintaining a relevant transfection efficiency.

## 1. Introduction

Recently, mRNA-based vaccines have attracted a lot of attention. Two mRNA vaccines, namely BNT162b2 (developed by Pfizer, New York, NY, USA, in collaboration with BioNtech, Mayence, Germany) and mRNA-1273 (from Moderna, Cambridge, MA, USA, in collaboration with the National Institute of Allergy and Infectious Diseases, Bethesda, MD, USA), reached Phase 3 clinical trial and were authorized for emergency use against SARS-CoV-2 infection by health authorities [1,2,3]. Their complete approval would make them the world’s first mRNA-based vaccines authorized for use on humans. For a long time, the emphasis was on the development of DNA-based vaccines (due to concerns associated with mRNA instability, inefficient in vivo delivery, high innate immunogenicity, and large-scale production limitations); however, recent technological innovations have made mRNA a realistic, promising, highly potent tool for vaccination [4]. If mRNA-based therapies are now a preferred research focus, it is also because they have many advantages over other approaches. Firstly, mRNA is not infectious (unlike whole virus delivery) and cannot integrate into the host’s genome or alter it (which may be a risk with DNA, which needs to reach the nucleus to be decoded) [5,6]. Moreover, mRNA natural degradation is processed within the organism, ensuring that mRNA activity is only temporary [5].

However, unprotected mRNA is highly unstable under physiological conditions because of its sensitivity toward catalytic hydrolysis by nucleases [7]. It is not efficiently internalized in its free form, mainly due to its large size (300–5000 kDa; 1–15 kB) [8,9] and negative charge [10,11]. In fact, it has been speculated that the cellular uptake rate and cytoplasmic transfer of naked mRNA is less than 1 in 10,000 molecules of the initial mRNA input [8,12]. As a result, the biggest hurdle to clinical approval of mRNA vaccines is the development of a delivery system that would allow mRNA to cross cell membranes without being degraded in order to be properly translated into antigenic proteins [5,7,13,14]. To date, numerous carriers have been described for this precise purpose, including dendrimers [15], polyethylenimine (PEI) [16,17], protamine [4], histidin/arginin-rich amphipathic peptides [6,18,19], and classical cationic liposomes. While the latter have been regarded as very attractive from an efficacy perspective, they have also been associated with toxicity and immunogenicity in vitro and in vivo [5,20]. Thus, lipid-based carriers, namely ionizable lipid nanoparticles (LNP), remain a mainstay of non-viral mRNA delivery systems [5,6,7]. Initially developed for siRNA delivery, they represent the most widely used in vivo mRNA delivery materials at present. In fact, the two vaccines mentioned above—in route to becoming the first prophylactic measures against Covid-19—actually rely on this type of carrier. In addition to a charged or ionizable lipid, LNP formulations typically include cholesterol, phospholipids and polyethylene glycol-lipids (PEG-lipids) [5,14] such as DMG-PEG 2000 [21] or C14-PEG 2000 [22].

Lipid-based nanocarriers can still be improved, both in terms of physicochemical characteristics and transfection efficiency. In this respect, we hypothesized that one way to do so would be to incorporate a solid core within a lipid membrane to ensure an efficient or synergistic uptake when compared to lipids alone. These assemblies—called LipoParticles (LP)—have already been described as interesting and versatile tools, combining the advantages of lipid membranes (bio-inspired properties) and nondeformable supports (ensuring mechanical stability) such as poly(lactic acid) nanoparticles (PLA-NP) [23,24,25]. With respect to the surface of LP, the use of lipids allows the final assemblies to interact with numerous species—including nucleic acids—as already shown for the adsorption of DNA plasmids onto LP-based PLA particles [26]. We focused our efforts on the use of PLA-NP, which are promising candidates in the field of vaccination, and which have been developed within our team for two decades [10,19,27]. PLA is a biopolymer which is receiving more and more attention, particularly because it is both biodegradable and biocompatible; its high safety profile has allowed it to be approved by the US Food and Drug Administration for diverse applications [28].

To test our hypothesis, we decided to evaluate the added value of the combination of PLA-NP and lipid coating (membranes made of 85/15 mol/mol 1,2-dioleoyl-3-trimethylammonium-propane (DOTAP)/1,2-distearoyl-sn-glycero-3- phosphocholine (DSPC)) in vitro through transfection experiments. To reach this goal, our strategy was based on a particulate layer-by-layer (pLbL) approach, using a successive adsorption of mRNA and a cell-penetrating peptide on the LipoParticles. Thus, our aim was to (i) design an innovative mRNA delivery platform based on LipoParticles (lipid-coated PLA nanoparticles), (ii) use our previous work on the vectorization of mRNA by PLA-NP and LAH4-L1 fusogenic peptides to potentiate the efficacy of the carriers [10], (iii) characterize and assess cytotoxicity and transfection efficiency of the final formulations on two different cell lines, and (iv) compare LipoParticles and liposomes alone.

## 2. Materials and Methods

### 2.1. Materials

The zwitterionic lipid 1,2-distearoyl-sn-glycero-3-phosphocholine (DSPC) and the cationic lipid 1,2-dioleoyl-3-trimethylammonium-propane (DOTAP) were purchased from Avanti Polar Lipids. (Alabaster, AL, USA). LAH4-L1 peptides (KKALLAHALHLLALLALHLAHALKKA) were purchased from GenScript (Piscataway, NJ, USA). The sterile pyrogen-free bidistilled water OTEC^®^ was purchased from Aguettant (Lyon, France) and the nuclease-free water from Ambion (via Thermo Fisher Scientific, Waltham, MA USA). Absolute ethanol came from Carlo Erba Reagents (Peypin, France). DPBS 1× and 10× (Dulbecco’s phosphate-buffered saline, pH 7.4), RPMI/glutamax and DMEM culture media, fetal bovine serum, β-mercaptoethanol, *N*-2-hydroxyethylpiperazine-*N*-2-ethane sulfonic acid) (HEPES), glutamine, penicillin/streptomycin and trypsin solution (0.25% Trypsin-EDTA (Ethylenediamine Tetraacetic Acid, Disodium Salt) were all purchased from Gibco (Dublin, Ireland).

### 2.2. Methods

#### 2.2.1. PLA-NP Synthesis

PLA-NP were provided by the company Adjuvatis (Lyon, France), using a surfactant-free solvent diffusion method (also called the nanoprecipitation technique) previously described [29]. Briefly, PLA polymer was dissolved in acetone and this solution was added dropwise to an aqueous solution made of ethanol and carbonate buffer under stirring. Once the nanoprecipitation was complete, some pyrogen-free water was added to assist the evaporation of the organic solvents under reduced pressure and controlled temperature using a Rotavapor R-300 (Buchi, Villebon sur Yvette, France). The solid content of suspensions was measured by weighing the wet and dry masses of the materials. The final suspensions were found to be stable for at least six months when stored at 4 °C.

#### 2.2.2. Microfluidic Production of Liposomes

Liposomes with 15/85 mol/mol DSPC/DOTAP molar formulation were manufactured using a NanoAssemblr™ benchtop instrument (NanoAssemblr™, Precision Nano-Systems Inc., Vancouver, BC, Canada) equipped with a microfluidic cartridge. The lipid solution (10 mM) was prepared by dissolving lipids in absolute ethanol. The total flow rate of the organic phase and water solution was fixed according to manufacturer’s recommendations. The liposomes were subsequently purified by dialyzing against 1× phosphate-buffered saline (PBS) at pH 7.4 using a Slide-A-Lyzer^®^ Dialysis cassette G2 (3 mL, MWCO 10kDa, Thermo Scientific™, Waltham, MA, USA) for at least 6 h. All liposomes were stored at 4 °C until use.

#### 2.2.3. LipoParticle Formulation from PLA-NP and Liposomes

LipoParticles were synthesized by incubating the preformed liposomes and PLA-NP in water solution. Briefly, liposomes were added to PLA-NP after adequate dilution and stirred using an orbital mixer (mLab scientific HCM 100-pro) for 1 h at 70 °C. The suspension was then centrifuged at 15 °C for 15 min at 4000× *g* in order to remove unadsorbed lipids. Thereafter, the supernatant was discarded and the LP-containing pellet was resuspended in the same volume of nuclease-free water. Note that LP were always prepared with an excess of liposomes compared to particles. This excess—defined in terms of liposomes’ surface area in relation to the NP surface area and noted Av/Ap—has already been well-described by our partners [23,25,30,31].

#### 2.2.4. Adsorption of Nucleic Acids and Cationic Cell-Penetrating Peptides Using pLbL Formulation

The pLbL approach consisted in the successive adsorption of nucleic acids and cationic LAH4-L1, mainly by means of electrostatic interactions. eGFP mRNA (CleanCap^®^ eGFP mRNA—(L-7601)—1 kB) and Fluc mRNA (CleanCap^®^ Fluc mRNA—(L-7602)—1.9 kB) were purified, optimized and purchased from TriLink BioTechnologies (San Diego, CA, USA). RNAs were quantified by spectrophotometric analysis at 260 nm and analyzed by standard agarose gel electrophoresis to confirm the integrity of the full-length mRNA. pcDNA™3.1 (Addgene plasmid # 18964), used to construct the two plasmids, was a gift from William Kaelin (Department of Medical Oncology, Dana-Farber Cancer Institute, Brigham and Women’s Hospital, Harvard Medical School, Boston, MA, USA). Plasmid pcDNA™3.1-luciferase was derived from fireflies and optimized for mammalian expression with the CMV promoter. pcDNA3.1-Luciferase (7 kB) encoding luciferase was kindly given by J.Y Exposito (LBTI, Lyon, France). All nucleic acids were stored in nuclease-free water at −20 °C. For the formulations, mRNAs or plasmids, diluted at a concentration of 40 ng/µL in nuclease-free water, were mixed (*v*/*v*) with LP or liposomes with a lipid concentration of 10mM, respectively. Then, two volumes of LAH4-L1 peptides (concentration adapted to the desired final mRNA/peptide ratio) were added to get the final pLbL formulations (either Liposomes-Ac Nuc-Peptides or LP-Ac Nuc-Peptides).

#### 2.2.5. Size and Zeta Potential Measurements

The average hydrodynamic diameter and the polydispersity index (PDI) of liposomes and LP, as well as final formulations, were measured by dynamic light scattering (DLS) at a scattering angle of 173° at 25 °C using a Zetasizer Nano ZS Plus (Malvern Instruments, Malvern, UK). The electrophoretic mobilities (zeta potential) of the same samples were determined by laser Doppler velocimetry using the same device. Prior to measurements, all samples were diluted (1/25) in filtrated 1 mM NaCl solution. Each value was the average of 4 measurements and three or four independent batches.

#### 2.2.6. Gel Retardation Assay

The complexation of mRNA within formulations was assessed using a gel retardation assay for electrophoresis. Agarose gel (1%) was prepared in Tris–borate–EDTA (TBE) (1×) buffer-containing ethidium bromide staining (EtBr, Genesee Scientific, San Diego, CA, USA). Samples were mixed with 2× loading dye (2× solution of 95% formamide, 18 mM EDTA, and 0.025% SDS, xylene cyanol, and bromophenol blue; Invitrogen, Carlsbad, CA, USA) and a volume corresponding to 100 ng (or equivalent volume for controls) of mRNA was loaded in each well. The electrophoresis process was run for 17 min at 100 V and the gel was observed in UV-Visible.

To dissociate mRNA from vectors and check its denaturation, a treatment was performed on samples prior to deposition. Briefly, formulation samples were treated—firstly with heparin (Sanofi-Aventis, Ploermel, France) for 1 h at RT, and secondly with proteinase K (NEB, Evry, France) for 30 min at 56 °C. Samples were then loaded into the gel and analyzed as described above.

#### 2.2.7. Cell Culture

Immortalized DC2.4 (a murine bone marrow-derived dendritic cell line) were obtained from InvivoGen (Toulouse, France) and cultured in RPMI-1640 medium, supplemented with 10% heat-inactivated FBS, 10 mM Hepes and 50 μM β-mercaptoethanol. HeLa cells were obtained from InvivoGen (Toulouse, France) and propagated in DMEM containing 10% heat-inactivated FBS only. Both cell lines were cultured in a 37 °C incubator (Heracell 150i, Thermo Scientific) under 5% CO_2_ and 95% humidity. Cells were always used with a low passage number (less than 10 and 20 for DC2.4 and HeLa, respectively).

#### 2.2.8. Transfection

DC2.4 and HeLa cells were seeded in a 96-well plate at a density of 20,000 cells (in 100 μL of complete medium) per well. After 24 h, the complete medium was replaced by 100 μL of serum-free medium containing the volume of formulations allowing the transfection of 90 ng (or equivalent volumes for controls) of either luciferase mRNA/pcDNA or eGFP mRNA/pcDNA. The supernatant was removed 3 h later and 100 μL of complete medium was added. Cells were then incubated at 37 °C under 5% CO_2_ and 95% humidity until the analysis (24 h later). Positive control was performed with Lipofectamine 2000 transfection reagent (Invitrogen™ via Thermo Scientific™, Waltham, MA, USA).

#### 2.2.9. Luciferase Assay

Transfection was carried out as described above with either mRNA-encoding luciferase or luciferase pcDNA3.1. Luciferase assay was performed 24 h later using the Bright-Glo™ Luciferase Assay System (Promega, Charbonnières-les-Bains, France) according to the manufacturer’s instructions. Briefly, 100 μL of Bright-Glo™ Luciferase assay substrate was added (*v*/*v*) per well. After 5 min at RT, the luminescence was detected on a Tecan i-control Infinite M1000 (Integration time 1 s) (Tecan, Männedorf, Switzerland). Luminescence was determined as the mean of three replicates and three independent experiments.

#### 2.2.10. Fluorescent Microscopy (eGFP)

Transfection was carried out as described above with either mRNA-encoding eGFP or Hyg-eGFP pcDNA3.1. 24 h later, cells were imaged and fluorescence excited with a motorized TiE Nikon inverted fluorescence microscope (Eclipse Ti-E, Nikon, Amsterdam, The Netherlands). eGFP fluorescence and phase contrast images were taken with a 10× objective.

#### 2.2.11. Cytotoxicity

Cytotoxicity of formulations was assessed using PrestoBlue™ Cell Viability Reagent (Invitrogen by Thermo Scientific™, Waltham, MA, USA) according to the manufacturer’s instructions. Briefly, transfection was performed as previously described and cytotoxicity was measured 24 h later. To do so, 10 μL per well of PrestoBlue™ Cell Viability Reagent was added and plates were incubated 20 min at 37 °C. Fluorescence was detected on Tecan i-control Infinite M1000 (560 nm/590 nm; Bandwidth 10 nm; optimal gain varying from 82% to 97%) (Tecan, Männedorf, Switzerland). Fluorescence was determined as the mean of three replicates and three independent experiments.

#### 2.2.12. Statistical Analysis

Statistical analyses were performed using GraphPad Prism version 8.0 software (San Diego, CA, USA). All of the data are presented as the mean ± SD where *n* = 3. Differences between groups were analyzed as described in figure legends. Statistical significance was indicated in the figures. A value of *p* < 0.01 was considered statistically significant.

## 3. Results

### 3.1. Study of the Added Value of a Solid Core within Liposomes

#### 3.1.1. Complexation of mRNA and LAH4-L1 on the Lipid Vectors Using the pLbL Approach

**Figure 1:**
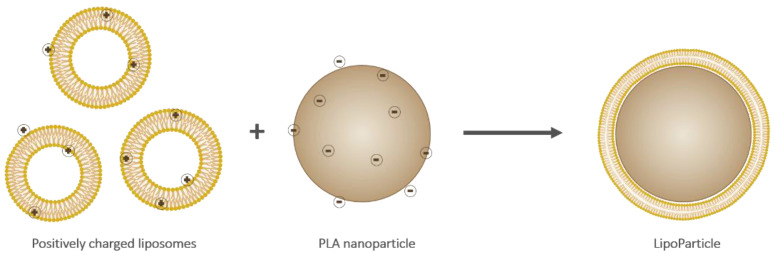
Schematic representation of cationic liposomes (**left**), an anionic poly(lactic) acid (PLA) nanoparticle (**middle**), and a resulting LipoParticle (**right**). Note that the scale and charge proportions are not respected.

Liposomes and LipoParticles (depicted in Figure 1) were prepared as described in the experimental section. LP were obtained by an adhesion and a reorganization of liposomes onto the PLA-NP surface.

After characterization by zetametry (Table 1), both vectors showed a positive surface charge (+45 mV and +58 mV for liposomes and LP respectively) due to the presence of 85% mol DOTAP in the formulation (a prerequisite criterion for complex mRNA through electrostatic interactions). The mean hydrodynamic diameter, measured by DLS, was different from one vector to another. LP were three times bigger than liposomes (245 nm for LP versus 81 nm for liposomes). In terms of size dispersion, LP suspensions were found to be more homogeneous than liposomes, with a polydispersity index (PDI) < 0.15. Interestingly, an inversion of the zeta potential charge was observed from anionic PLA-NP to cationic LP due to the particulate surface modification by cationic liposomes.

**Figure 2:**
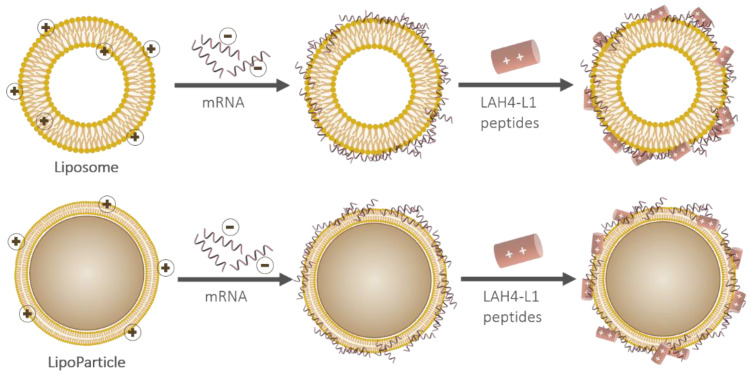
Schematic representation of the particulate layer-by-layer formulation strategy used to successively adsorb mRNA and LAH4-L1 on either a liposome (**top**) or a LipoParticle (**bottom**). Note that the scale proportions are not respected.

A pLbL strategy was then implemented (Figure 2). This was accomplished via the immobilization of mRNA and the cationic peptide LAH4-L1 on the surface of the vectors. As the main goal of our work was to evaluate the in vitro capacity of these assemblies to deliver any mRNA and to express a functional protein in eukaryotic cells, we initially focused on the adsorption of mRNA-encoding luciferase enzyme.

The different steps of the pLbL strategy were individually characterized by DLS and zetametry (Table 2). The main parameter of interest was the surface charge, representative of an efficient adsorption (or not) of the two layers (negatively charged mRNA and positively charged LAH4-L1) on the carriers.

As expected, the surface charge underwent a first inversion following the addition of the Fluc mRNA on the LipoParticles to reach a negative value of −38 mV. This zeta potential was again reversed after the addition of the second layer (LAH4-L1 peptides), suggesting the adsorption of LAH4-L1 on the intermediates [LP + mRNA]. Surprisingly, the surface charge of the 15/85 mol/mol DSPC/DOTAP liposome formulation remained the same after the additions of mRNA and cationic peptides. The mean hydrodynamic diameter and PDI increased during the pLbL formulation compared to the initial naked vectors. This was consistent with the adsorption of molecules on their surface.

**Figure 3:**
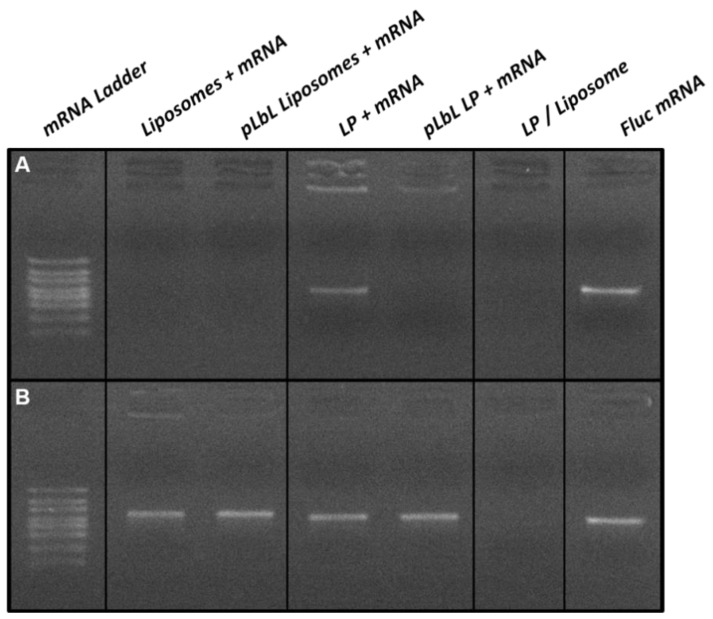
Agarose gel electrophoresis analysis of mRNA-based formulations (**A**) without and (**B**) with mRNA desorption treatment. For desorption treatment, samples were incubated at room temperature for 30 min and then treated with heparin (to desorb mRNAs and peptides) and proteinase K (to degrade peptides) at 56 °C for 15 min.

The complexation of mRNA through the pLbL strategy was also assessed by agarose gel electrophoresis as shown in Figure 3A. While the mRNA was fully complexed to the liposomes at each step of the formulation process, part of it remained in free form when formulated with the LP only (i.e., without the addition of LAH4-L1 peptides). This migration of free mRNA was no longer visible with the pLbL approach, reflecting the fact that the process enabled the total complexation of mRNA on LP.

A second agarose gel electrophoresis, shown in Figure 3B, was carried out using the same formulations; however, an mRNA desorption treatment using heparin and proteinase K was performed prior to gel deposition in order to verify the integrity of the mRNA. The heparin treatment allowed the desorption of mRNAs and peptides from the surface of the vectors, while the proteinase K was used to degrade the peptides. In the presence of this treatment, all chemical bonds will be disrupted, and mRNA—if not degraded—will migrate within the gel in the same way as free mRNA. This was observed for all the formulations studied, and this clearly evidenced that the pLbL formulation allowed the adsorption of mRNA without degradation.

#### 3.1.2. Fluc mRNA Transfection Capacity and In Vitro Cytotoxicity of Each Formulation

The ability of the two vectors of interest (liposomes and LP) to transfect cells was evaluated in vitro using murine dendritic cells (DC 2.4) and immortalized human epithelial cells (HeLa). The different steps of the pLbL formulation approach were individually controlled in order to highlight the interest of such a process.

**Figure 4:**
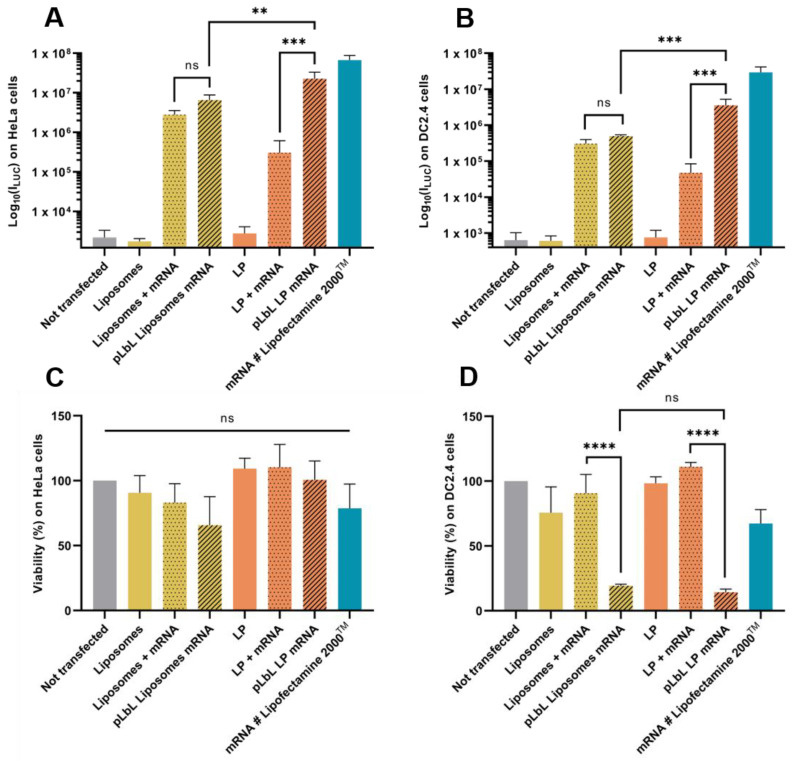
In vitro evaluation at +24 h (top) of transfection efficiency through measurement of luminescence intensity (Bright-Glo luciferase assay) and (bottom) cell viability (PrestoBlue assay) obtained after transfection of 90 ng *eq.* of Fluc mRNA, formulated either in liposomes or in LP using pLbL strategy (ratio mRNA/LAH4-L1 = 1:20 *w*/*w*) on HeLa (**A**,**C**) and DC 2.4 (**B**,**D**) cells. Lipofectamine 2000^TM^ was used as positive control. Data are presented as mean ± SD and statistically analyzed using one-way ANOVA followed by Tukey’s multiple comparison test (not significant (ns): *p* > 0.01, **: *p* < 0.001, ***: *p* < 0.0001 and ****: *p* < 0.00001).

As shown in Figure 4A,B, both liposomes and LP enabled efficient Fluc mRNA expression in HeLa and DC 2.4 cells. However, LP have been shown to induce significantly higher transfection efficiency (compared to liposomes) with the pLbL approach, regardless of the cell type chosen. While the formulations allowed for adequate expression of mRNAs within cells with or without the addition of LAH4-L1, the addition of the latter led to a positive overall charge and significantly increased transfection efficiency (factor > 70) with LP as carrier (Figure 4B). This highlighted the interest of the particulate layer-by-layer formulation strategy, which potentiated the mRNA vectorization and expression. However, the response tends to be a bit lower than with gold standard lipofectamine 2000^TM^.

Although the transfection profiles were very similar on the two cell types studied, differences appeared when looking at cell viability. None of the naked vectors or formulations were toxic to HeLa cells, compared to cells alone (Figure 4C). However, when looking at DC 2.4, it was observed that the addition of LAH4-L1 peptide in the formulations systematically and drastically decreased cell viability (both with liposomes and LP) (Figure 4D).

#### 3.1.3. eGFP mRNA Transfection of Lipid Formulations

**Figure 5:**
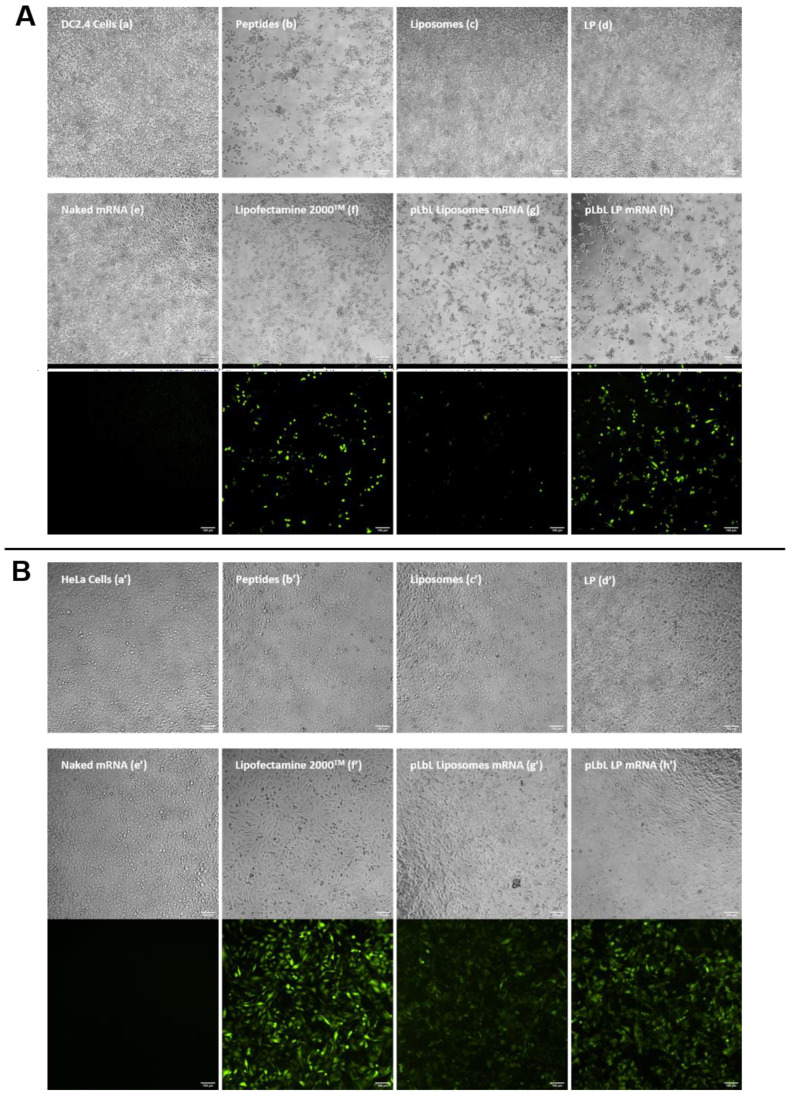
In vitro assessment of (top images) the cytotoxicity (normal phase mode) and (bottom images) transfection efficiency (fluorescent mode) of pLbL formulations using microscopy. Images taken 24 h after transfection of 90 ng *eq.* of eGFP mRNA, formulated either in liposomes or in LP using pLbL strategy (ratio mRNA/LAH4-L1 = 1:20 *w*/*w*) on DC 2.4 ((**A**), (**a**–**h**)) and HeLa ((**B**), (**a’**–**h’**)) cells. Lipofectamine 2000^TM^ was used as positive control. Scale bar: 100µm. (**a**,**a’**) Cells alone (**b**,**b’**) LAH4-L1 peptide (**c**,**c’**) Liposomes, (**d**,**d’**) LP (**e**,**e’**) Naked mRNA (**f**,**f’**) Lipofectamine 2000^TM^ (**g**,**g’**) pLbL Liposomes mRNA (**h**,**h’**) pLbL LP mRNA.

To confirm the results obtained in the previous section, the same experiment was carried out using another mRNA, namely mRNA encoding eGFP. The qualitative analysis (of transfection efficiency on the one hand, and cytotoxicity on the other) was carried out by optical/fluorescence microscopy; images are depicted in Figure 5.

The results were consistent with those obtained previously with mRNA-encoding luciferase. The naked vectors (c’,d’), peptide (b’), pLbL formulations (g’,h’), and lipofectamine (f’) were nontoxic compared to nontransfected HeLa cells (a’), with the cells remaining homogeneously distributed within the wells. As for DC 2.4 cells, liposomes (c), LP (d) alone, and naked mRNA (e) all showed no toxicity. The toxicity of the peptide, on the other hand, was clearly illustrated (b), with significant cell death observed close to the center of the wells, justifying the toxicity of the pLbL formulations which contained this peptide on their surface. As expected, naked mRNA did not transfect any cell line (e,e’), whereas pLbL formulations with LP allowed a very efficient transfection and expression of eGFP mRNA both on DC 2.4 and HeLa cells (h,h’), similar to those achieved with lipofectamine 2000^TM^ (f,f’). As previously observed, liposomes appeared to be less effective in transfecting cells (g,g’).

Among the two mRNA carriers, results showed that LP containing a PLA solid core were more efficient in vitro than liposomes when using a pLbL strategy (with equal toxicity). Thus, to extend these observations, we decided to focus our next studies on DNA delivery, and—for clarity reasons—have presented only the data with LP studies in the next section. However, the results with liposomes are available as supplementary data (Table S1, Figure S1).

### 3.2. Study of the Versatility of the LP as Carrier

To study the versatility of LP as a nucleic acid delivery tool, we prepared and evaluated pLbL formulations with DNA plasmid of 7 kB using the same approach. Briefly, luc pcDNA3.1 and LAH4-L1 were adsorbed sequentially on LP. Each nanoassemby (including intermediate steps) was tested on DC 2.4 and HeLa cells by transfection. As performed with mRNA, formulations were characterized with Zetasizer Nano ZS Plus, and the results are presented in Table 3.

The DNA formulations obtained were similar to those with mRNA, with two successive charge reversals during the pLbL process. The mean hydrodynamic diameter as well as the PDI were increased following the addition of the DNA. Only the PDI value was increased after adding the cationic peptide.

**Figure 6:**
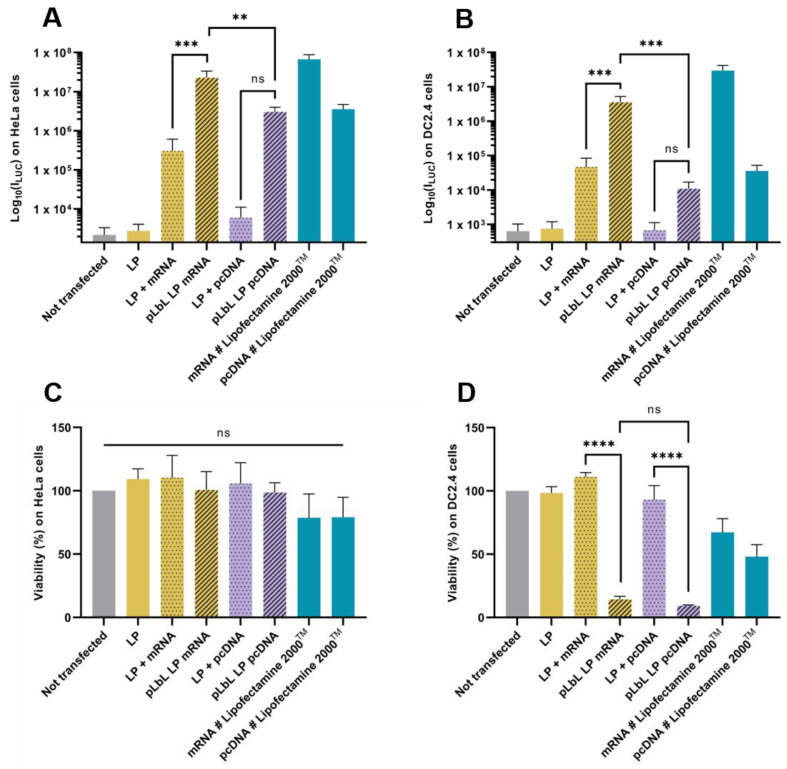
In vitro evaluation at +24 h (top) of transfection efficiency through the measurement of luminescence intensity (Bright-Glo luciferase assay) and (bottom) cell viability (PrestoBlue assay) obtained after transfection of 90 ng *eq*. of either Fluc mRNA or luciferase-pcDNA3.1, formulated in LP using pLbL strategy (ratio nucleic acids/LAH4-L1 = 1:20 *w*/*w*) on HeLa (**A**,**C**) and DC 2.4 (**B**,**D**) cells. Lipofectamine 2000^TM^ was used as positive control. Data are presented as mean ± SD and statistically analyzed using one-way ANOVA followed by Tukey’s multiple comparison test (ns: *p* > 0.01, **: *p* <0.001, ***: *p* < 0.0001 and ****: *p* < 0.00001).

For the transfection assay on each cell line (HeLa or DC2.4), each formulation—including mRNA as read out—was compared. In addition, lipofectamine 2000^TM^ was used as a positive control. Results are represented in Figure 6.

As depicted on the different graphs, the results obtained with DNA plasmids were very similar (in terms of results profile) to those obtained with mRNA, whatever the type of cells used. Thus, the pLbL approach allowed a more efficient expression of proteins of interest than the intermediate formulation LP-nucleic acids (without peptides). However, this improvement of transfection was associated with toxicity in DC 2.4 cells (Figure 6D), as noted with mRNA delivery. On the other hand, none of the formulations appeared to be toxic to HeLa cells, as also observed with mRNA (Figure 6C). Interestingly, transfection efficiency was significantly lower with DNA plasmid than mRNA (Figure 6A,B).

In summary, LP, when formulated with the pLbL strategy, are able to protect and deliver two types of nucleic acids (namely, mRNA and DNA plasmids) in two very different cell lines. Such results illustrate the high versatility of LP as nucleic acid (mRNA and DNA plasmids) delivery supports.

### 3.3. Optimisation Strategy to Reduce Cytotoxicity on Dendritric Cells of the pLbL Approach: Modification of the Nucleic Acids/Peptides Ratio

Although very promising, the pLbL approach is still limited, due to the apparent toxicity on DC 2.4, which is inherent to the LAH4-L1 peptide. To address this issue, we hypothetized that balancing the nucleic acid/peptide would decrease the cytoxocity of the final formulations on DC 2.4 cells while maintaining a correct level of expression of the proteins of interest.

**Figure 7:**
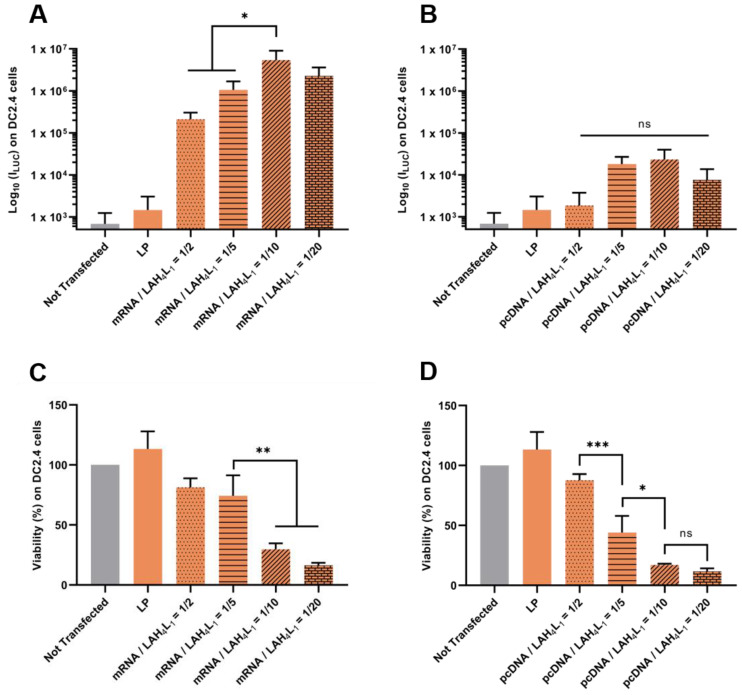
Evaluation of the influence of nucleic acids/ LAH4-L1 *w*/*w* ratio during pLbL formulation. Analysis of (top) the transfection efficiency by measuring luminescence intensity (Bright-Glo luciferase assay) and (bottom) cell viability (PrestoBlue assay) obtained after transfection of 90 ng *eq.* of either Fluc mRNA or luciferase-pcDNA3.1 formulated in LP using the pLbL strategy. Nucleic acids/LAH4-L1 *w*/*w* ratio ranged from 1:2 to 1:20. Transfection was performed both on HeLa (**A**,**C**) and DC 2.4 (**B**,**D**) cells. Lipofectamine 2000^TM^ was used as positive control. Data are presented as mean ± SD and statistically analyzed using one-way ANOVA followed by Tukey’s multiple comparison test (ns: *p* > 0.01, *: *p* < 0.01, **: *p* < 0.001 and ***: *p* < 0.0001).

Firstly, our results evidenced that it was possible to reduce the toxicity of pLbL formulations by adjusting the *w*/*w* mRNA/LAH4-L1 ratio (Figure 7C) or *w*/*w* pcDNA/LAH4-L1 ratio (Figure 7D). A pLbL strategy with an acid nucleic/peptide *w*/*w* ratio equal to 1:2 showed cell viability of 82% and 88% with mRNA and pcDNA, respectively. With a ratio of 1:20, the same respective cell viabilities were only 16% and 12%. While the adjustment of this ratio did not significantly change the transfection efficiency with the DNA plasmid (Figure 7B)—meaning that the 1:2 ratio could be used to deliver this type of nucleic acid—higher differences emerged for mRNA delivery. In this case, the pLbL formulation with an mRNA/LAHA-L1 *w*/*w* ratio of 1:10 allowed a significantly higher transfection than with the 1:2 and 1:5 ratios (but no significant difference with 1:20) (Figure 7A). However, the expression of Fluc mRNA remained still convincing in vitro, regardless of the ratio used, especially when compared to pcDNA expression. As a result, the strategy based on the optimization of the mRNA/peptide ratio to enable efficient delivery of mRNAs while reducing cytotoxicity seems promising.

## 4. Discussion

mRNA is a very promising approach for vaccination, and the approval of two Covid-19 mRNA vaccines by health authorities—both of which are lipid-based formulations—opens new opportunities in this field. However, obstacles remain which limit the performance of these vaccines, including the quantity of mRNA necessary. These obstacles exist due to the rapid degradation of naked mRNA by nucleases, but also the difficulty of the vectors carrying them to target antigen-presenting cells after vaccine administration. Furthermore, after entering the cell, mRNA vaccines need to escape from endosomes to ensure efficient translation of mRNA by cell machinery. In order to overcome these drawbacks, better mRNA delivery systems to the cytoplasm of the cells of interest need to be identified.

Optimization of these vectors through in vitro model screening is primordial and always necessary before considering any preclinical or clinical trial. In this paper, we sought to evaluate the influence of a polymeric solid core (PLA-NP) in lipid membranes for mRNA transport within cells using transfection experiments. Our group (Coolen AL) has previously shown that PLA nanoparticles were interesting for such a purpose. These PLA-NP, when combined with mRNA and LAH4-L1 cell penetrating peptides, demonstrated a strong ability to transfect dendritic cells (DCs) and induce strong protein expression [10]. On the other hand, the delivery systems of choice for mRNA remain those based on the use of lipids. From these two observations, we designed and compared LipoParticles to liposomes alone, which have often proven to be interesting but also very toxic [32,33].

Surfactant-free PLA nanoparticles were synthesized using a safe-by-design protocol. The absence of additives gave PLA-NP an interesting safety profile, in contrast to PLA suspensions usually described in the literature which, despite their efficacy, contain surfactants with potential toxicity on cells [34]. In order to maintain this added value, liposomes and LP were synthesized without any addition of surfactants. The production method currently used, although safe-by-design, is standardized and must be optimized before considering a large-scale application. While we have chosen to use automated microfluidics as a way of optimization, other production methods exist, particularly for liposomes [35,36].

All the vectors obtained had the expected characteristics in terms of size and zeta potential, with a mean hydrodynamic diameter below 250 nm and an overall positive charge—the latter being important for post-adsorption of negatively charged nucleic acids. In addition, the colloidal structure of LP has already been highlighted previously by TEM images [23,25]. The formulation of these vectors with mRNA and LAH4-L1 worked with the pLbL strategy, which consisted in using electrostatic interactions (and to a certain extend hydrophobic interactions and hydrogen bonding) [37] to adsorb the mRNAs on (first) either 15/85 mol/mol DSPC/DOTAP liposomes or PLA coated with 15/85 mol/mol DSPC/DOTAP lipid membranes (LP), and (second) the peptides. While this was clearly demonstrated with the LP by the two successive inversions of surface charge (a first one to reach a negative value following mRNA adsorption and a second one to return to a final positive charge after the addition of the peptides), the surface charge of the 15/85 mol/mol DSPC/DOTAP liposome formulation remained the same after the addition of both mRNA and cationic peptides. Several hypotheses could explain such observations: (i) mRNA and peptides remained in free form in the formulation; (ii) mRNA has not been added in sufficient quantity to lower the surface charge; or (iii) a rearrangement of the lipids took place during the addition and the mRNAs became trapped in the lipid matrix. The first hypothesis was quickly ruled out following the performance of an agarose gel electrophoresis to examine the binding capacity of mRNA to cationic vector. Results showed that not only was the mRNA fully adsorbed in the formulations (with liposomes or LP), it was also not degraded by the pLbL process.

The formulations obtained through the pLbL strategy, whether with liposomes or LP, allowed the efficient transfection of the two cell types used as models here—namely, DC 2.4 dendritic cells and HeLa epithelial cells. Several studies have already shown that positively charged delivery systems lead to much greater cellular internalization than neutral or negatively charged systems [5]; this explains why the addition of the LAH4-L1 peptide in each case resulted in significantly higher mRNA expression than with vector + mRNA systems only. As the latter are characterized by a negative surface charge, interactions with similarly-charged cell membranes are unfavorable to internalization. In addition, as mRNA delivery materials are too large to readily diffuse across cell membranes and are therefore usually taken up into cells by endocytosis [38], the translation of mRNA into proteins can only occur if the vesicles manage to escape the endosome that separate them from their site of action (in this case, the cytosol). However, while endosomal escape is essential to elicit an appropriate therapeutic response, the process may be the most challenging aspect. It has been estimated that world-class RNA delivery materials manage to escape the endosome only about 2% of the time [5,39]. To overcome this issue, several strategies may be considered. In a recent article, Sabnis and her partners from Moderna developed a series of novel amino lipids that improved endosomal escape of mRNA in primates by achieving 2–15% release in the cytosol—depending on the lipid used [40]. Cell-penetrating peptides (also called endosomal escape peptides or EEPs) such as LAH4-L1 have emerged as a promising tool, and, in this case, played an important role in the transfection process using pLbL formulations [41]. Indeed, in addition to being positively charged (and thus allowing spontaneous electrostatic interactions—first with negatively charged phosphate backbone of nucleic acids [42] and then with anionic lipid membranes), LAH4-L1 peptide possesses pH-sensitive residues that facilitate endosomal escape [38,43]. While the exact mechanism of action is still unclear, endosomal release of histidine-rich peptides is thought to occur by buffering against the adenosine-5′-triphosphate (ATP)-dependent proton pump located in the endosome membrane. As protons are pumped in, the imidazole group of histidine—which has a pKa of ~6.0 and therefore can be protonated in acidic media of the endosome—adsorbs protons, leading to endosomal swelling and destabilization and then membrane disruption [43]. To support this proton sponge theory, plasmid DNA transfection efficiency of cells by LAH4 peptides was significantly reduced when endosomal acidification was inhibited by the H+-ATPase inhibitor bafilomycin A1. This indicates that protonation of the imidazole groups of histidine residues is important for endosomal lysis—and thus for efficient transfection [44,45].

However, while these arguments make LAH4-L1 a very useful component in the pLbL strategy, this peptide has also been found to be toxic within [vector + mRNA] + LAH4-L1 formulations on phagocytic DC 2.4 cells, whereas it was not on nonphagocytic HeLa cells. Legaz et al. demonstrated a relationship between cell viability and the uptake of NPs by cells, with a significant decrease in cell viability when there is a high accumulation of NPs within cells [46]. It can therefore be postulated that LP covered with mRNA and LAH4-L1 accumulate more in DC 2.4 than in HeLa cells (due to DC2.4 cells’ high capacity for engulfing particles), thus increasing cytotoxicity in DC 2.4 cells. As toxicity of cell-penetrating peptides has already been highlighted in several articles [19,47], a preliminary study was conducted in this article to reduce the effects of LAH4-L1 on DC 2.4 cells. By decreasing the mRNA/LAH4-L1 *w*/*w* ratio in the pLbL formulation from 1:20 to 1:5, cytotoxicity was divided by a factor larger than 3 without altering the transfection efficiency.

Both liposomes and LP appeared to be an efficient tool for delivering mRNA into the cytosol using the pLbL approach. However, the incorporation of a solid core made of PLA nanoparticles in lipid membranes significantly improved transfection compared to liposomes alone on both cell types taken as models. This is reflective of the importance of considering physical characteristics when developing a delivery system. In this particular case, the rigidity of the cargo appears to have a significant impact on cell transfection. Liposomes are often limited by their intrinsic instability and are prone to fuse with each other, leading to payload loss [48]. One can then postulate, following Troutier’s observations, that the solid core acts as a cytoskeleton which confers a mechanical stability on the lipid layers that reduces fusion—and thus reduces undesired mRNA loss prior to transfection [31]. In fact, rigidity has already been qualified as an important criterion for the development of immunization systems, reinforcing the idea of the added value of a PLA-NP to improve the efficiency of liposomes as vectorization materials, especially when considering an application in the field of vaccination. Mazumdar et al. demonstrated that increasing the rigidity of the lipid bilayer led to an increase in the stability of liposomes encapsulating *leishmania donovani* antigens compared with those composed of more ‘fluid’ lipids and thus enabled the triggering of humoral and cell-mediated immune responses in vivo. [49]. In another study, DDA (dimethyldioctadecylammonium) rigid liposomes were found to be preferred as a vaccine delivery system, as they were more potent inducers of both humoral and cellular immune responses than their unsaturated analog DODA (dimethyldioleoylammonium) liposomes [50,51].

Our in vitro transfection data also highlighted the versatility of LP, since they were able to vectorize both mRNA and DNA. Moreover, they allowed the adsorption of two mRNAs (eGFP and Fluc) of different sizes (996 bases and 1921 bases, respectively), making it possible to consider their use in vaccination with larger mRNA-encoding antigenic proteins such as the spike protein of coronavirus (4 kb) or HIV-1 glycoproteins (1.8 kb). This versatility has a definite advantage; it could allow for easy and rapid adaptation of the vector for different nucleic acids in the long term. However, these results must be confirmed both on primary cells and on in vivo models before any real application in vaccination can be envisaged.

## 5. Conclusions

In this paper, we sought to evaluate (in vitro) the value of a solid core in liposomes when considering a vaccine application. Adsorption of mRNA onto the two vectors of interest was performed by the pLbL strategy, which consisted in using mainly electrostatic interactions to successively adsorb mRNA and a cell-penetrating peptide (LAH4-L1) onto the surface of LP. LP allowed much greater transfection than liposomes, and the results were validated with two different mRNA and DNA. On the other hand, interest in the pLbL formulation strategy was highlighted, as the strategy potentiated mRNA vectorization and expression by allowing the delivery of mRNA into the cytosol through a facilitated endosomal escape.

## Figures and Tables

**Table 1:** Characterization of poly(lactic) acid nanoparticles (PLA-NP), 15/85 mol/mol 1,2-distearoyl-sn-glycero-3-phosphocholine (DSPC)/1,2-dioleoyl-3-trimethylammonium-propane (DOTAP) liposomes and LipoParticles (LP). Results are represented as mean ± SD of different batches (*n* = 3 for PLA-NP, *n* = 4 for liposomes and LP).

Type of Vector	Mean Hydrodynamic Diameter (nm)	Polydispersity Index (PDI)	Zeta Potential (mV)
NP-PLA (for LP elaboration)	151 ± 3	0.094 ± 0.013	−64 ± 3
Liposomes (for LP elaboration)	81 ± 3	0.215 ± 0.009	45 ± 4
LipoParticles	245 ± 14	0.135 ± 0.007	58 ± 4

**Table 2:** Characterization of Fluc mRNA adsorbent formulations prepared through particulate layer-by-layer (pLbL) strategy using either liposomes or LP as carriers (ratio mRNA/LAH4-L1 = 1/20 *w*/*w*). Results are represented as mean ± SD of different batches (*n* = 3).

Formulation	Mean Hydrodynamic Diameter (nm)	Dispersity (PDI)	Zeta Potential (mV)
Liposomes	81 ± 3	0.215 ± 0.009	45 ± 4
Liposomes-mRNA	108 ± 4	0.370 ± 0.048	43 ± 5
Liposomes-mRNA-LAH4-L1 (pLbL)	113 ± 1	0.283 ± 0.082	44 ± 2
LP	245 ± 14	0.135 ± 0.007	58 ± 4
LP-mRNA	260 ± 27	0.229 ± 0.037	−38 ± 7
LP-mRNA-LAH4-L1 (pLbL)	305 ± 16	0.269 ± 0.026	44 ± 2

**Table 3:** Characterization of luc pcDNA formulations prepared through pLbL strategy using LP as carrier (ratio pcDNA/LAH4-L1 = 1/20 *w*/*w*). Results are represented as mean ± SD of different batches (*n* = 3).

Formulation	Mean Hydrodynamic Diameter (nm)	Dispersity (PDI)	Zeta Potential (mV)
LP	245 ± 14	0.135 ± 0.007	58 ± 4
LP-pcDNA	307 ± 21	0.264 ± 0.039	−37 ± 6
LP-pcDNA-LAH4-L1 (pLbL)	306 ± 4	0.286 ± 0.013	37 ± 1

## Data Availability

Not applicable.

## Supplementary Materials

The following are available online at https://www.mdpi.com/1999-4923/13/3/377/s1: Table S1. Characterization of luc pcDNA formulations prepared through pLbL strategy using liposomes as carrier (ratio pcDNA/LAH4-L1 = 1/20 *w*/*w*). Results are represented as mean ± SD of different batches (*n* = 3); Figure S1. In vitro evaluation at +24 h (top) of the transfection efficiency through the measurement of the luminescence intensity (Bright-Glo luciferase assay) and (bottom) cell viability (PrestoBlue assay) obtained after transfection of 90 ng eq. of either Fluc mRNA or luciferase-pcDNA3.1 formulated in liposomes using pLbL strategy (ratio nucleic acids/LAH4-L1 = 1:20 *w*/*w*) on HeLa (A,C) and DC 2.4 (B,D) cells. Lipofectamine 2000^TM^ was used as positive control. Data are presented as mean ± SD and statistically analyzed using one-way ANOVA followed by Tukey’s multiple comparison test (not significant (ns): *p* > 0.01, **: *p* < 0.001, ***: *p* < 0.0001 and ****: *p* < 0.00001).
