# Supplementary material for: LipoParticles: Lipid-Coated PLA Nanoparticles Enhanced In Vitro mRNA Transfection Compared to Liposomes
Source: Pharmaceutics. 2021 Mar 12;13(3):377. doi: 10.3390/pharmaceutics13030377 (PMC7999670; doi:10.3390/pharmaceutics13030377)
Supplement: Supplementary file 1 [file pharmaceutics-13-00377-s001.pdf]

pharmaceutics

MDPI

Supplementary Materials: LipoParticles: Lipid-Coated PLA Nanoparticles Enhanced In Vitro mRNA Transfection Compared to Liposomes

Camille Ayad, Pierre Libeau, Céline Lacroix-Gimon, Catherine Ladavière and Bernard Verrier

Study of the transfection efficiency of pcDNA with liposomes as carrier

Table S1. Characterization of luc pcDNA formulations prepared through pLbL strategy using liposomes as carrier (ratio pcDNA/LAH4-L1 = 1/20 *w/w*). Results are represented as mean ± SD of different batches (*n* = 3).

Formulation	Mean hydrodynamic diameter (nm)	Polydispersity index (PDI)	Zêta potential (mV)
Liposomes	81 ± 3	0.215 ± 0.009	45 ± 4
Liposomes-pcDNA	108 ± 4	0.370 ± 0.048	43 ± 5
Liposomes-pcDNA-LAH4-L1 (LbL)	95 ± 3	0.228 ± 0.019	47 ± 1

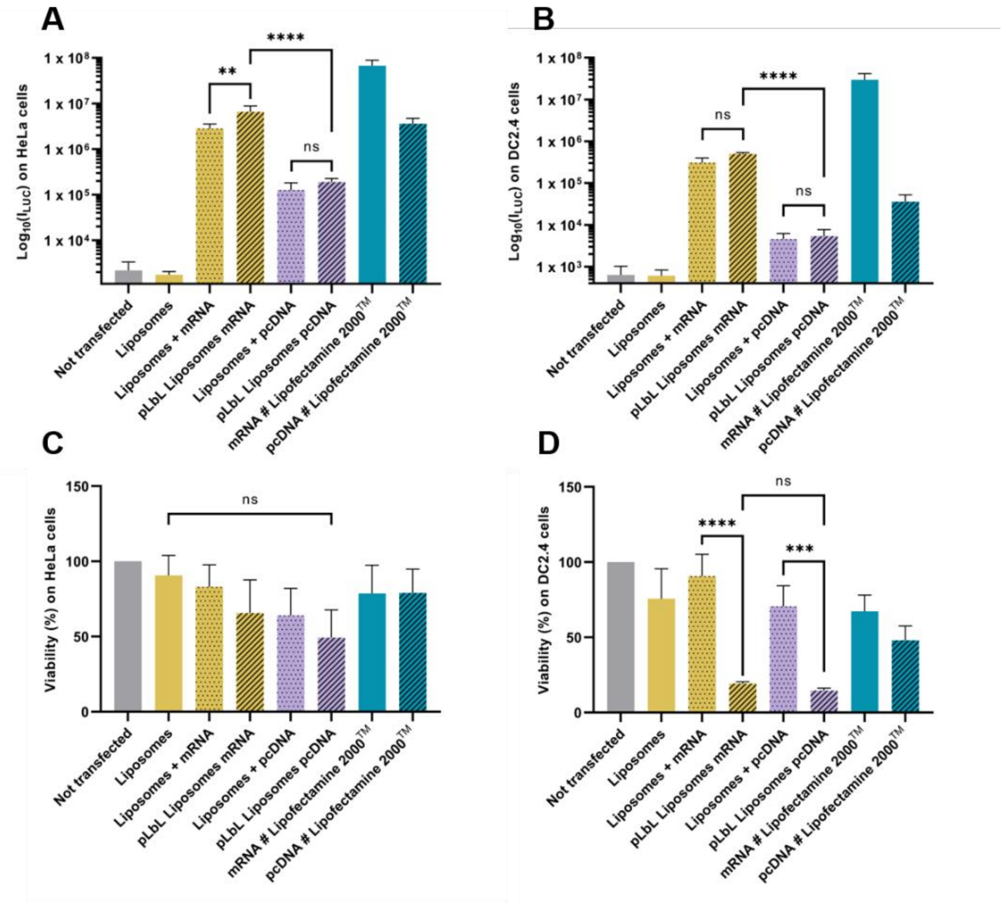

Figure S1. In vitro evaluation at +24 h (top) of the transfection efficiency through the measurement of the luminescence intensity (Bright-Glo luciferase Assay) and (bottom) cell viability (Presto-Blue assay) obtained after transfection of 90 ng eq. of either Fluc mRNA or luciferase-pcDNA3.1 formulated in liposomes using pLbL strategy (ratio nucleic acids/LAH4-L1 = 1:20 *w/w*) on HeLa **(A,C)** and DC 2.4 **(B,D)** cells. Lipofectamine 2000™ was used as positive control. Data are presented as mean ± SD and statistically analyzed using one-way ANOVA followed by Tukey's multiple comparison test (not significant (ns): $p > 0.01$, **: $p < 0.001$, ***: $p < 0.0001$ and ****: $p < 0.00001$).
